# Strategic global data integration to improve genomic prediction accuracy in tree breeding programs facing resource limitations, a case study in mango

**DOI:** 10.1093/hr/uhag004

**Published:** 2026-01-06

**Authors:** Abdulqader Jighly, Norman Munyengwa, Reem Joukhadar, Vanika Garg, Natalie Dillon, Rhys G R Copeland, Jugpreet Singh, Sukhwinder Singh, Christopher I Cazzonelli, Penghao Wang, Peter Prentis, Craig Hardner, Rajeev K Varshney

**Affiliations:** State Agricultural Biotechnology Centre, Centre for Crop and Food Innovation, Food Futures Institute, Murdoch University, Murdoch, WA, 6150, Australia; AgriSapiens PTY LTD, VIC 3108, Australia; Queensland Alliance for Agriculture and Food Innovation, The University of Queensland, Brisbane, Queensland 4072, Australia; AgriSapiens PTY LTD, VIC 3108, Australia; State Agricultural Biotechnology Centre, Centre for Crop and Food Innovation, Food Futures Institute, Murdoch University, Murdoch, WA, 6150, Australia; Queensland Department of Primary Industries, Mareeba, Queensland 4880, Australia; State Agricultural Biotechnology Centre, Centre for Crop and Food Innovation, Food Futures Institute, Murdoch University, Murdoch, WA, 6150, Australia; Department of Horticultural Sciences, University of Florida IFAS, Tropical Research and Education Centre, Homestead, FL 33031, USA; United States Department of Agriculture, Agricultural Research Service, Subtropical Horticulture Research Station (SHRS), Miami, FL 33158, USA; Hawkesbury Institute for the Environment, Hawkesbury Campus, Western Sydney University, Richmond, New South Wales 2753, Australia; State Agricultural Biotechnology Centre, Centre for Crop and Food Innovation, Food Futures Institute, Murdoch University, Murdoch, WA, 6150, Australia; Centre for Agriculture and Bioeconomy, Queensland University of Technology, Brisbane, Queensland 4001, Australia; Queensland Alliance for Agriculture and Food Innovation, The University of Queensland, Brisbane, Queensland 4072, Australia; State Agricultural Biotechnology Centre, Centre for Crop and Food Innovation, Food Futures Institute, Murdoch University, Murdoch, WA, 6150, Australia

## Abstract

Genomic prediction (GP) in mango breeding faces challenges due to the species’ complex biology, long cycles, and limited reference populations. To accelerate genetic improvement, this study integrated data from diverse global populations to increase the reference population size. It included three mango collections reserved in Australia (225), USA (161), and China (224), totaling 610 individuals. Fruit weight (FW) and total soluble solids (TSS) were measured in multiple datasets, while several other traits were measured in specific datasets. We evaluated genetic diversity, performed genome-wide association studies (GWAS), and assessed GP accuracy using standard, genotype-by-environment (GxE), and multitrait models, both within and across collections. Findings revealed a highly admixed genetic structure, with faster linkage disequilibrium (LD) decay in the Chinese collection, indicating higher genetic diversity. Data integration significantly enhanced GWAS power, identifying 19 quantitative trait loci (QTLs) for FW and 9 for TSS. GxE models consistently achieved higher or comparable prediction accuracies for FW and TSS compared to the non-GxE models, especially when combining Australian and US collections. This was not the case when predicting into or from the Chinese collection, mostly due to differences in the phenotyping protocol. While single-trait models performed comparably to multitrait models in predicting new individuals (Cross-Validation: CV1), multitrait models significantly improved prediction accuracy in scenarios with incomplete phenotypic records (CV2). This study demonstrates that strategic global data integration significantly enhances GWAS power and GP accuracy in mango. This collaborative approach is crucial for developing more efficient and accelerated breeding programmes for mango and other perennial trees.

## Introduction

Mango (*Mangifera indica* L.), a globally significant tropical fruit tree, holds of considerable economic and nutritional importance [1]. The continuous genetic improvement of mango varieties is crucial for ensuring global food security and addressing the challenges posed by climate change and evolving consumer demands [2]. Accelerated breeding programmes are essential to develop new cultivars with enhanced yield, superior fruit quality, and increased resilience to biotic and abiotic stresses [3]. Therefore, adopting new technologies and the latest modern breeding strategies is critical to achieve future production goals.

Genomic prediction (GP), which uses genome-wide molecular markers to estimate the breeding value of individuals, offers a powerful tool to accelerate selection and genetic gain in breeding programs [4]. However, the application of GP in tree crops, including mango, lags behind annual crops due to biological and agronomic challenges [5, 6]. These include a long juvenile phase and extended generation times, typically spanning 5–9 y before fruiting and overall breeding cycles spanning 15–20 y, which significantly delay trait evaluation and cultivar release [7, 8]. Furthermore, mango exhibits high heterozygosity and complex reproductive biology, characterized by polyembryony, low crossing rates, and single-seed fruits [9]. Moreover, the substantial land, labor, and financial investments required for maintaining large-scale experimental orchards constrain the size and scope of reference populations [10, 11]. Additional challenges include limited knowledge of the trait heritability and a potential reduction in genetic diversity among elite commercial cultivars, which can directly impair breeding efficiency.

Beyond these biological constraints, significant bottlenecks arise from phenotyping complexity and resource limitations. Phenotypic data collection in mango remains largely manual, labor-intensive, and time-consuming [12], limiting the size and quality of the collected data for robust reference populations for GP. Many complex traits in mango are significantly influenced by environmental factors and genotype-by-environment interactions (GxE), which introduce noise into phenotypic data and complicate the accurate assessment of genetic potential [13]. However, GxE for different traits in mango is largely unexplored [14]. These technical difficulties are aligned with resource and infrastructure constraints, including insufficient funding and investment in plant genetic resource conservation, which limits the characterization and accessibility of diverse mango germplasm.

The cumulative effect of these challenges is a significant barrier to the accurate and efficient implementation of GP in mango. The combination of limited resources, challenges in collecting high-quality phenotypic data over long periods, and the inherent genetic complexity leads to smaller, less representative reference populations. This causes lower prediction accuracy of genomic estimated breeding values (GEBVs) and limits the reproducibility of GP models to broader breeding efforts [15]. Consequently, the pace of genetic gain remains slow, and the development of improved mango varieties continues to be a slow and resource-intensive process.

To explore the full potential of GP for overcoming these challenges in mango, a strategic and collaborative approach is required [16]. This includes integrating phenotypic and genotypic data from multiple research resources and genetically diverse germplasms across different institutes and geographical locations [17]. Such an integrated approach would facilitate the establishment of larger, more diverse, and more robust reference populations [18], thereby enhancing the accuracy and broader applicability of GP models and accelerating the development of superior mango cultivars.

Effective global data integration is crucial for improving the accuracy of GP in trees. Here, this importance is exemplified through a case study focused on mangoes. We investigated the utility of integrating three mango germplasm collections from Australia, the USA, and China, for which phenotype data was available for fruit weight (FW), and the first two were phenotyped for total soluble solids (TSS). Additional traits were measured independently within each germplasm collection. The present study aimed to evaluate (i) the genetic diversity across the three germplasm collections; (ii) the power of the genome-wide association study (GWAS) using the integrated collection; (iii) the improvement in the accuracy of GP within each germplasm collection and in the integrated collection using standard, GxE, and multitrait GP models.

## Results

### Genetic diversity and linkage disequilibrium

**Figure 1:**
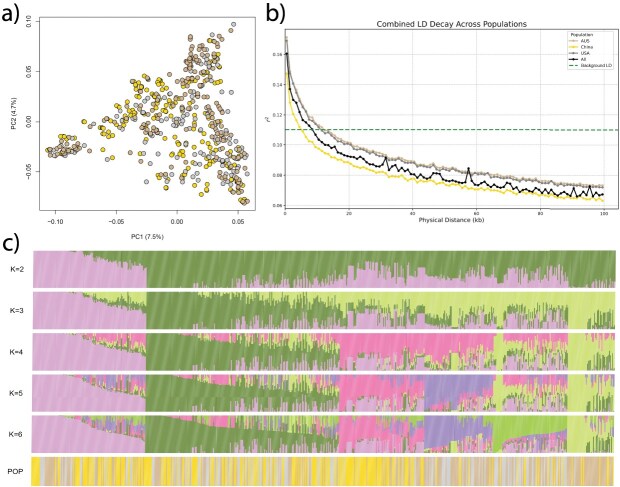
Genetic diversity across the three studied mango collections. (a) PCA illustrating the distribution of the three collections on the first two principal components. (b) LD decay for each of the three individual collections, as well as for all collections combined. (c) Population structure showing genetic clusters ranging from *K* = 2 to *K* = 6. The bottom row in panel (c) indicates the specific collection to which each individual belongs.

The single nucleotide polymorphism (SNP) calling initially yielded 14 656 007 SNPs that were reduced to 12 520 922 high-quality SNPs after further filtering across the 610 samples, which were used for subsequent downstream analyses. The analysis of genetic diversity, utilizing principal component analysis (PCA) and ADMIXTURE across the integrated dataset of 610 mango accessions, indicated a highly admixed population structure. The PCA plot (Fig. 1a) did not reveal a clear separation among the Australian, Chinese, and US collections, suggesting substantial admixture. The first principal component (PC1) explained 7.5% of the total genetic variation, while the second principal component (PC2) explained 4.7% of the variation. The ADMIXTURE analysis (Fig. 1c) confirmed a high admixture across the three collections. However, within the six inferred subpopulations (*K* = 6), one subpopulation (subpopulation 3, pink; Table S1) was predominantly derived from the Chinese collection, and another (subpopulation 6, light green; Table S1) was mainly from the Australian collection. Two other subpopulations (subpopulations 4 and 5, purple and green; Table S1) primarily originated from a mix of Australian and US accessions. The remaining two subpopulations were highly admixed across the three subpopulations.

The background linkage disequilibrium (LD) analysis determined as the 99th percentile of unlinked SNP pairs revealed the *r^2^* threshold of 0.11. The LD decay of *r^2^* values with increasing genetic distance varied among the three collections (Fig. 1b). The Australian and US collections exhibited a very similar LD decay pattern, both falling below the critical *r^2^* threshold at ~15 kb. In contrast, the Chinese collection showed a faster LD decay, dropping below the threshold at ~7 kb. When considering all collections combined, the overall LD decay reached the threshold at ~10 kb, falling in the middle across all collections.

### SNP-based heritability and genetic correlation

**Figure 2:**
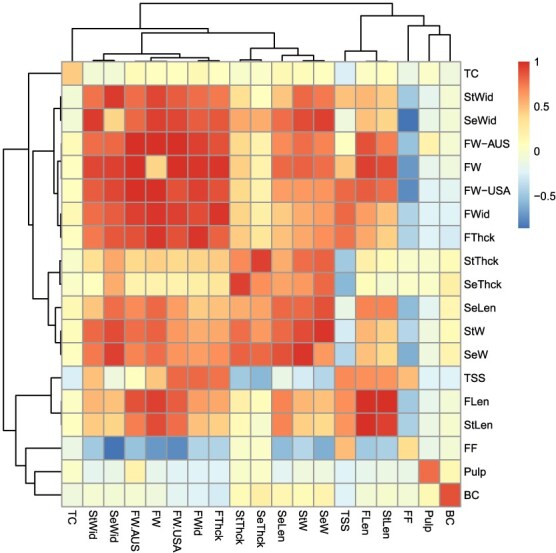
Genetic correlation-based clustering of the studied traits across the three studied mango collections. The diagonal represents the heritability. Detailed values can be found in Table S2a. Blush color (BC), fruit firmness (FF), trunk circumference (TC), total soluble solids (TSS), pulp percentage (Plup), fruit length (FLen), fruit thickness (FThck), fruit weight (FW), fruit width (FWid), stone length (StLen), stone thickness (StThck), stone weight (StW), stone width (StWid); seed length (SeLen), seed thickness (SeThck), seed weight (SeW), and seed width (SeWid).

The estimated SNP-based heritability values (*h^2^*) and genetic correlations among the studied traits are presented in Fig. 2 and summarized in Table S2a. The *h^2^* values ranged between 0.11 for FW in the Chinese collection to 1 ± 0.05 for fruit length in the US collection. The average *h^2^* value across all traits was equal to 0.71. Overall, many fruit quality and morphological traits exhibited high SNP-based heritability values, suggesting strong genetic control over their variation within the studied collections. For instance, FW consistently showed high heritability across the Australian and the US collections (0.88–1.00 ± 0.05), indicating that most of its variation is genetically determined. TSS also displayed a high heritability of 0.70, but trunk circumference (TC) showed moderate heritability (0.45), suggesting greater environmental influence compared to fruit traits.

Genetic correlations revealed complex relationships among the traits (Fig. 2; Table S2a). These genetic correlations showed comparable values to the phenotypic correlation values (Table S2b) with slightly higher absolute values in general. FW in the Chinese collection was excluded from this analysis because it had a very low *h^2^* of 0.11, which was lower than its standard deviation (SD = 0.14) leading to unreliable estimation of genetic correlation. As expected, highly positive genetic correlations were observed among different fruit, seed, and stone dimension traits, including weight, thickness, length, and width. Interestingly, fruit firmness (FF) had a negative correlation with this cluster of traits. TSS showed a strong positive genetic correlation with fruit traits but a negative one with seed and stone traits. TC, blush color (BC), and pulp showed generally low genetic correlations with all other traits.

### Genome-wide association study

**Figure 3:**
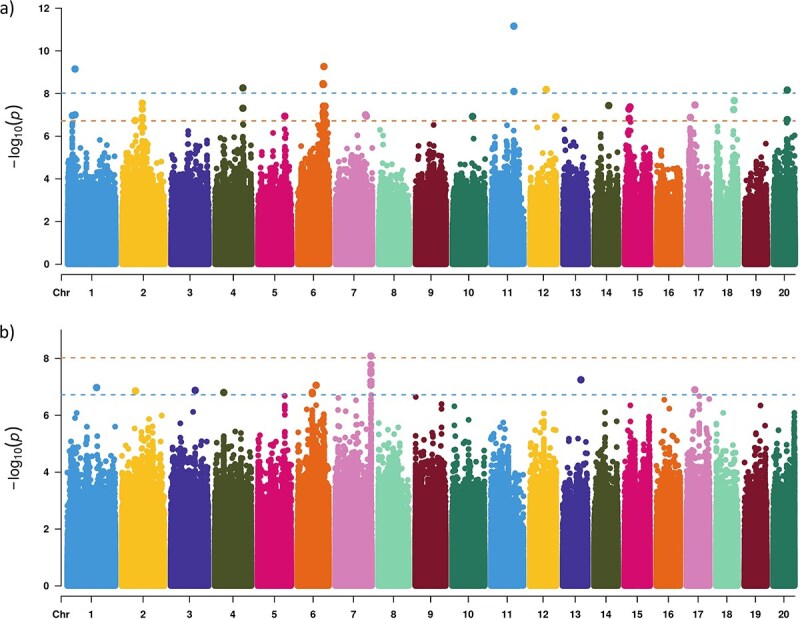
Manhattan plots showing the GWAS results from the integrated dataset for (a) FW and (b) TSS traits. The top horizontal line represents the significant threshold, while the lower one represents the suggestive association threshold.

The GWAS for FW and TSS in the integrated mango dataset identified multiple significant QTLs. The results are visualized in Fig. 3 and detailed in Table S3, while QQ plots can be found in Fig. S1. The Genetic Type 1 Error Calculator (GEC) method revealed that the independent number of SNPs was equal to 5 235 150 SNPs (41.8% of all SNPs). Therefore, using Bonferroni correction, the GEC method estimated the significant threshold at 9.5 × 10^−9^ and the suggestive association at 1.9 × 10^−7^ as revealed by the GEC method. For FW, a total of 44 significant SNPs that can be clustered on 19 distinct QTLs were detected across chromosomes 1, 2, 4, 5, 6, 7, 10, 11, 12, 14, 15, 17, 18, and 20 (Table S3). Six out of the 19 QTLs showed highly significant associations, while the remaining 13 QTLs were considered as suggestive associations. For TSS, a total of 14 significant SNPs, clustered on nine QTLs, were identified on chromosomes 1, 2, 3, 4, 6, 7, 13, and 17 (Table S3). Of these QTLs, only one QTL on chromosome 7 showed a highly significant association, while the remaining 6 QTLs were considered as suggestive associations. The candidate genes surrounding the FW QTLs are functionally linked to key processes controlling fruit size including receptor-like kinases; genes associated with cell proliferation; and enzymes essential for biomass allocation and structural component synthesis. For TSS, the detected QTL is linked to a gene annotated as calcium-dependent protein kinase (Table S4).

### Genomic prediction

GP accuracy was evaluated for all traits using the single-trait model. Given that the traits FW and TSS were recorded in multiple collections, they were also analyzed using the EG and GxE models. The cluster of morphological traits that showed a high positive correlation (Fig. 2) was further analyzed using the multitrait (MT) and the multitrait with interaction (MxT) models. The prediction accuracy for the remaining traits that were only analyzed using the single-trait model can be found in Table S5.

For FW, prediction accuracies varied considerably depending on the reference population used. Compared to the single trait/environment model, including the Chinese collection in the reference population had a negative effect on predicting the performance in the other two collections under all evaluated scenarios (Table 1). Moreover, the prediction accuracy for within the Chinese collection was significantly lower than the other two collections in all scenarios. High prediction accuracies were consistently observed when validating in the Australian and US collections, regardless of whether the reference population comprised two or three collections (Table 1). In these cases, the GxE model consistently yielded slightly higher or comparable accuracies compared to the EG model. However, compared to the single-trait analysis, the only scenario that showed significantly higher prediction accuracy was when applying the GxE model on a reference population comprising the Australian and the US collections. On the other hand, for TSS, the prediction accuracy for the single-trait model was comparable to that of the GxE model and they both were significantly higher than the prediction accuracy of the EG model.

**Table 1:** The accuracy of GP within reference populations of FW and TSS traits

**Trait**	**Reference**	**Validation**	**Single**	**EG**	**GxE**
**FW**	**AUS-CHN**	**AUS**	0.71	0.65	0.67
		**CHN**	0.20	0.14	0.14
	**AUS-USA**	**AUS**	0.71	0.72	0.74
		**USA**	0.60	0.66	0.65
	**CHN-USA**	**CHN**	0.20	0.14	0.16
		**USA**	0.60	0.52	0.59
	**All**	**AUS**	0.71	0.70	0.71
		**CHN**	0.20	0.15	0.17
		**USA**	0.60	0.61	0.63
**TSS**	**AUS-USA**	**AUS**	0.61	0.39	0.60
		**USA**	0.59	0.41	0.60

Accuracies are presented for the ‘single’ (single-trait), ‘EG’ (all environments), and ‘GxE’ (genotype-by-environment interaction) GP models. The analysis evaluates prediction using either two or three collections for reference and validation sets. In this table, the reference and validation environments are the same. Standard deviations across all tested scenarios over the 50 replicates ranged between 0.07 and 0.11.

For a cross-collection prediction scenarios, accuracies were generally higher when predicting between the Australian and US collections (Table 2). For instance, using the Australian collection as a reference resulted in a prediction accuracy of 0.63 for the US validation population, while the US reference predicted the Australian collection with an accuracy of 0.60. However, prediction into the Chinese validation collection was consistently very poor, with accuracies of 0.04 and 0.03 when using Australian or US collections as reference, respectively. Furthermore, when the Chinese population was used as a reference, it provided no predictive power for the Australian or US validation collections (accuracy of 0.0). Combining two collections as a reference resulted in moderate prediction accuracies for Australian (0.54) and US (0.48) validation populations, which again reflects the negative effect of the Chinese population on predicting the performance in the other two collections. Nevertheless, prediction accuracy for the Chinese validation collection remained very low (0.07) even with a reference from the remaining two collections was used.

**Table 2:** GP accuracy for FW in intercollection prediction scenarios, when using one or two reference populations to predict each validation population

**Reference**	**AUS**	**USA**	**CHN**
**AUS**	0.71	0.63	0.04
**USA**	0.60	0.60	0.03
**CHN**	0.00	0.00	0.20
**2 Collections**	0.54	0.48	0.07

For the ‘2 collections’ scenario, each pair of collections was used as a reference to predict the third one (column name).

The accuracy of multitrait (MT and MxT) GP models, leveraging highly correlated traits such as the widths, lengths, and weights of fruit, seed, and stone, as well as fruit thickness, are presented in Table 3. For the cross-validation (CV)1 scenario, the multitrait models (MT and MxT) generally did not outperform the ‘single’ trait/environment model. In many cases, their accuracies were slightly lower, suggesting that for complete data scenarios, leveraging trait correlations through multitrait models may not always provide a substantial advantage over single-trait prediction. However, a dramatically different pattern was observed in the CV2 scenario. For almost all traits, the multitrait models, particularly MxT, showed substantial improvements in prediction accuracy compared to the ‘single’ model. The average prediction accuracy across all traits for the single-trait analysis was 0.55. This average significantly increased to 0.66 for the MT model and further to 0.72 for the MxT model in the CV2 scenario.

**Table 3:** The accuracy of GP using the multitrait (MT and MxT) models that utilized the highly correlated traits

**Trait**	**Single**	**CV1**		**CV2**	
		**MT**	**MxT**	**MT**	**MxT**
**FW-AUS**	0.71	0.59	0.57	0.58	0.55
**FW-USA**	0.60	0.57	0.54	0.87	0.83
**SeWid**	0.35	0.33	0.38	0.54	0.57
**SeW**	0.42	0.32	0.41	0.59	0.67
**SeLen**	0.41	0.34	0.37	0.62	0.69
**StW**	0.45	0.37	0.46	0.62	0.71
**StWid**	0.57	0.45	0.55	0.67	0.72
**StLen**	0.66	0.55	0.66	0.67	0.79
**FWid**	0.62	0.44	0.60	0.66	0.80
**FThck**	0.60	0.44	0.59	0.64	0.80
**FLen**	0.66	0.56	0.63	0.77	0.81

These traits are the widths, lengths, and weights of the fruit, seed, and stone, as well as the fruit thickness. Accuracies are presented for two CV scenarios (CV1 and CV2).

Fruit length (FLen), fruit thickness (FThck), fruit weight (FW), fruit width (FWid), stone length (StLen), stone weight (StW), stone width (StWid); seed length (SeLen), seed weight (SeW), and seed width (SeWid)

## Discussion

Initiating a genomic-guided breeding program in fruit tree crops like mango requires the establishment of a robust population to serve as a reference for prediction. However, the assembly, long-term management, and data collection on such tree populations are inherently challenging due to financial constraints, extended generation times, and logistical barriers [10]. As a result, tree breeding programs often maintain small, diverse populations within individual orchards [11]. This study aimed to address these limitations in tree GP by integrating phenotypic and genotypic data from three geographically diverse global mango germplasm collections from Australia, China, and the USA. We assessed the impact of this data integration on genetic diversity, the power of GWAS, and the accuracy of GP models both within and across collections. Our findings revealed a highly admixed genetic structure across the integrated mango collections, with varying linkage disequilibrium decay rates. We successfully identified multiple significant QTLs for key fruit traits, and we demonstrated that incorporating GxE interactions significantly improved GP accuracies, highlighting the practical utility of a collaborative, data-integrated approach for enhancing genetic analyses and accelerating breeding outcomes in mango.

Our analysis of genetic diversity revealed a highly admixed population structure across the integrated Australian, Chinese, and US mango accessions. This is expected given that the three germplasm collections are not native to these countries but are independent collections from all over the world. The admixture level and genetic diversity reported here seems to be higher than most previously reported studies [19–24], which is expected given the international scope of the integrated germplasm. The observed LD decay patterns varied among the collections. While the Australian and US collections exhibited similar LD decay, the Chinese collection displayed a noticeably faster decay. This more rapid LD decay in the Chinese collection indicates a higher level of underlying genetic diversity within this germplasm compared to the Australian and US collections. This enhanced diversity is critical, suggesting that the Chinese collection could be a valuable resource for introducing novel genetic variation into the more genetically uniform Australian and US breeding programs. Such a source of new alleles is essential for broadening the genetic base, enhancing resilience to emerging biotic and abiotic threats and facilitating the development of new cultivars with improved traits, thereby mitigating potential reductions in genetic diversity among elite commercial cultivars [25]. On the other hand, the LD decay when using the combined collection showed slower decay compared to the Chinese collection alone, which could result from the population structure, although small, between this collection and the other two collections (Fig. 1).

The estimated *h^2^* values revealed strong genetic control over many fruit quality and morphological traits within the studied collections. However, FW in the Chinese collection showed a very low *h^2^* and was excluded from genetic correlation analyses. This result is likely a consequence of the specific phenotyping methodology employed, where FW was measured as the maximum weight from a random sample of at least 15 fruits per tree, rather than an average [26]. While this approach might reflect consumer preference for large fruit at the point of sale, it is less optimal for accurately capturing the genetic inheritance of the trait. Mango trees typically exhibit significant within-tree variation in fruit size due to factors such as within-tree phenology variation, fruit set position, branch load, and microenvironmental differences [12, 27, 28]. Relying on the maximum size from limited, randomly sampled fruits make it challenging to consistently represent the genetic potential of a cultivar, as it is unlikely that the ‘top tier’ of fruit (e.g. the largest 5%) would be sampled uniformly across all measured accessions. An average fruit weight generally provides a more robust and heritable estimate that better reflects the genetic contribution to the trait, thus improving the accuracy of genetic parameter estimations and GPs. We would like to emphasize that these results are not biased due to the differences in the genetic covariances or scaling among different collections given that the GxE model applied here consider these factors.

The genetic correlations among traits provided further insights into their underlying genetic architecture. Strong positive genetic correlations were observed among various fruit, seed, and stone dimension traits, indicating pleiotropic genetic effects influencing overall fruit development, which is consistent with previous reports [29–31]. Genetic correlations showed very similar trends to the phenotypic correlations with generally higher absolute values, potentially due to excluding the environmental noise. Interestingly, FF was negatively correlated with this cluster of fruit size traits. This may reflect a trade-off where increased fruit size and associated water accumulation could lead to less rigid cell wall structures, resulting in softer fruit at ripe maturity, but further research is required to confirm that. On the other hand, TSS was positively correlated with overall fruit size, similar to previous reports [30]. Positive correlation of TSS with overall fruit size suggests that sugar accumulation is linked to the general growth of the entire fruit, with the fruit acting as a predominant sink for sugar import [32]. Conversely, the negative correlation between seed/stone dimensions and TSS might result from a resource allocation trade-off, where larger reproductive structures might reduce the available resources in the flesh. However, future research should investigate that.

The population size is one of the main factors affecting the power of the GWAS analysis [33]. The reanalysis of FW and TSS using the integrated dataset significantly enhanced the power of GWAS and led to the identification of several highly significant QTLs. This contrasts sharply with the original individual studies [26, 34, 35], in which the Australian and the US data did not report significant QTLs at a stringent genome-wide significance threshold, relying instead on suggestive association thresholds. The present study detected six highly significant associations for FW, and one for TSS, by leveraging the significantly larger combined collection size (610 individuals). This demonstrates the increased GWAS power, highlighting the advantage of data integration, as a larger sample size improves the statistical power to detect associations between genetic markers and phenotypic traits [36]. These findings not only provide critical insights into the genomic regions influencing key fruit traits in mango but also validate the strategic approach of combining disparate datasets to overcome the limitations in individual, smaller studies in perennial tree crops.

A crucial methodological decision in our GWAS was setting the significance threshold. While the Bonferroni correction provides high confidence and minimizes false positives for the reported QTLs, applying it based on the total number of SNPs is often excessively stringent and leads to a high rate of false-negative outcomes. To mitigate this risk, we corrected only for the effective number of independent SNPs. This method accounts for LD, resulting in a less conservative, more appropriate significance threshold [37]. Despite this methodological refinement to improve statistical power, we acknowledge that the stringent nature of any Bonferroni-based correction means that QTLs with genuinely small effects may still fall below the detection threshold. For this reason, we provided a list of suggestive associations as recommended by the GEC method that we used to calculate the independent number of SNPs [37]. Therefore, the limited number of QTL detections along with the population size could be interpreted within this context, recognizing that the genetic architecture of these traits may involve a larger number of loci than currently reported. Genetic variation in FW is primarily controlled by the rate of cell division and the extent of cell expansion [38]. Here, we identified candidate genes central to nucleotide synthesis and structural component production. Dihydroorotase is key to pyrimidine *de novo* biosynthesis, a pathway critical for generating the building blocks of DNA and RNA. Since pyrimidine availability can directly limit the rate of cell proliferation [39], its association with an FW QTL suggests that genetic variation at this locus influences the speed or capacity for cell multiplication in the developing fruit, thus regulating final biomass [40]. Supporting the requirement for structural components, hydroxymethylglutaryl-CoA synthase-like is a key regulator of the mevalonate (MVA) pathway, which is essential for producing sterols and brassinosteroid hormones critical for cell expansion. Furthermore, the MVA pathway is critical to terpenoid and aroma synthesis in mango [41, 42], directly affecting fruit quality and growth.

Other FW QTLs are positioned near genes that regulate the perception of environmental and hormonal signals that control growth and survival. The identification of a leucine-rich repeat receptor-like serine/threonine-protein kinase (LRR-RLK) and a receptor-like protein kinase 5 (RLK5) highlights a critical layer of growth signaling control. These receptor-like kinases are master regulators that perceive internal and external signals to directly dictate cell division and expansion [43]. RLK5 is a critical player in several developmental pathways, including those governing cell expansion and mediating responses to hormones such as abscisic acid (ABA) [44]. Also, the DEAD-box ATP-dependent RNA helicase genes, known for their critical role in post-transcriptional gene regulation and fruitlet abscission in mango [45], and the NDR1/HIN1-like protein, associated with stress response, are also identified near FW QTLs. The colocalization of molybdenum cofactor sulfurase-like further links growth to hormonal regulation, as it mediates the synthesis of the molybdenum cofactor, which is required for enzymes in the ABA synthesis pathway [46].

Genetic control over fruit quality and biomass is further mediated by the efficient allocation of photosynthates. The identification of a sugar phosphate/phosphate translocator (SPPT) near a FW QTLs highlights the importance of carbon resource partitioning. The colocalization of RLK5 (an ABA-related kinase) and the SPPT is biologically significant, as recent studies demonstrated that the ABA signaling pathway directly controls sugar transport modules essential for fruit ripening and biomass accumulation [47]. Genetic variation in the efficiency of this SPPT locus may therefore constrain final fruit biomass by limiting sugar delivery capacity to the developing fruit.

Finally, the genetic control over fruit quality is highlighted by the candidate gene for TSS, a Calcium-Dependent Protein Kinase (CDPK). This association is strongly supported by earlier mango-specific research demonstrating that calcium-regulated protein kinase activities are central to the fruit ripening process [48, 49]. CDPKs act as molecular switches that translate the crucial calcium signal into cellular responses, directly linking them to the complex regulatory network underpinning overall fruit quality [50]. For TSS, the CDPK gene likely controls metabolic shift towards sugar accumulation by regulating key carbohydrate-metabolizing enzymes during the final stages of ripening. These findings demonstrate that FW and TSS are controlled by the convergence of structural synthesis, resource allocation, and hormonal signaling pathways.

Integrating diverse global datasets is critical for successful GP in trees to address the inherent limitations of small breeding populations. This approach increases the effective population size and phenotypic diversity available for training GP models, which is essential for improving selection accuracy and optimizing future crosses [51–53]. A larger and more diverse reference population allows for the capture of a wider range of genetic variability and environmental adaptations, facilitating the development of climate-resilient cultivars [54, 55]. In trees like mango, characterized by long breeding cycles and resource-intensive phenotyping, such integration is required for accelerating genetic gain and achieving breeding objectives. Successful data integration, however, requires harmonizing genotyping and phenotyping data [56]. Our results suggest that while integrating diverse collections is powerful for successful intercollection GP, it requires careful consideration of shared genetic backgrounds and harmonized phenotyping methods. For example, the Chinese dataset reduced prediction accuracy for FW when combined with Australian or US data, likely due to methodological differences in trait measurement. Future efforts should focus on standardizing phenotyping across international germplasm collections and exploring more sophisticated models that can better capture complex genetic architectures and GxE interactions across highly divergent populations to fully utilize the potential of global data integration for GP in mango breeding. Moreover, the proposed approach here has the potential to extend its applications to other tree and crop species [57].

GxE represents a significant challenge and opportunity in GP, as it describes how the genetic expression of a trait can vary across different environments [58]. Accurately modelling GxE is essential for predicting site-specific performance and identifying genotypes that exhibit consistent performance or specific adaptation across diverse target environments [59–61]. Studies have consistently shown that GP models incorporating GxE components often yield higher prediction accuracies compared to univariate models, particularly in multienvironment trials or when phenotyping data are incomplete [62, 63]. For mango breeding, where cultivars are grown across varied climates and management practices, accounting for GxE interactions is crucial. Our results, which demonstrate improved prediction accuracies with GxE models, highlight the importance of these models in guiding selection decisions and optimizing germplasm development and deployment across the global mango production areas. Moreover, the level of GxE between the Australian and US collections was low enough to allow intercollection prediction, but still significant enough to improve the prediction accuracy.

The significant increase in prediction accuracy observed for the multitrait models under the CV2 strategy provides a powerful demonstration of the MT model’s utility in practical breeding programs. The CV2 strategy simulates the common real-world challenge of missing data, where an individual has been genotyped and observed for one or more traits, but is missing records for others. The substantial improvement here is driven by the MT model’s ability to leverage the underlying genetic correlations among traits. With the individual’s genetic profile fixed, the model can effectively borrow strength from the genetically correlated, observed traits to accurately impute the performance of the missing trait. For example, if a strong positive correlation exists between fruit length and fruit weight, the observation of a long fruit informs a more accurate prediction of its weight. This ability to fill in missing phenotypic data by relying on the trait’s shared genetic architecture is highly valuable, as it allows breeders to maximize the information gained from limited phenotyping efforts, thereby enhancing the selection efficiency for traits that are expensive, labor intensive, or destructive to measure on every single plant, using simpler correlated traits.

## Conclusion and future directions

This study demonstrates the potential of integrating diverse genomic and phenotypic datasets to accelerate mango breeding efforts, directly addressing the key limitations imposed by the tree’s long juvenile phase, complex reproductive biology, and resource-intensive breeding cycles. By combining datasets from Australia, China, and the USA, the improved GWAS power allowed for more precise identification of genetic loci controlling key traits, while enhanced GP accuracies for some traits/environments enabled more efficient selection of superior genotypes at earlier stages. These advancements can significantly reduce the time and cost of developing new cultivars with enhanced yield, superior fruit quality, and increased resilience to environmental stresses. While challenges exist, particularly in intercollection prediction due to phenotypic method inconsistencies, the overall gains in GWAS power and the robust performance of GP models under specific scenarios strongly support this collaborative approach. Overcoming the challenges will require ongoing efforts to harmonize phenotyping protocols and develop more advanced statistical models capable of better accounting for heterogeneity across datasets. Despite these complexities, the strategic integration of data from multiple research resources and diverse germplasm collections is a powerful strategy to establish larger, more robust reference populations, thereby maximizing the utility of genomic tools for mango improvement.

This collaborative approach directly addresses the resource limitations and biological complexities inherent in mango breeding, but also leverages the genetic diversity available globally, facilitating more targeted and efficient selection of superior cultivars. Future research should prioritize expanding and harmonizing phenotyping protocols across international collaborators, exploring advanced GP models to account for complex genetic architectures and environmental variability, such as integrating biophysical growth models with GP [64, 65], and developing high-throughput phenotyping methods. These include image analyses [66], near-infrared (NIR), and mid-infrared (MIR) spectroscopy [67, 68], and multiomics platforms including transcriptomic, proteomic, and metabolomic advances [50]. Such integrative efforts will be crucial to fully harness genomic technologies to develop improved mango varieties adapted to evolving agricultural demands and environmental challenges. Moreover, the framework presented here can be readily applied to other tree species facing similar biological and logistical limitations. By facilitating more efficient breeding strategies, this approach contributes to the development of resilient, high-performing cultivars, ultimately supporting global food security and the long-term sustainability of fruit production systems.

## Materials and methods

### Plant materials

Three distinct datasets from Australia, China, and the USA [26, 34, 35] were obtained and integrated for this study. These datasets collectively comprised 610 mango trees from diverse global origins: 225 from Australia, 224 from China, and 161 from the USA. A list of 20 individuals were common (clones) across the Australian and the US collections (Table S6).

### Australian data

Phenotypic and genotypic data for a total of 225 mango trees, originating from 24 different countries and maintained in the Australian Mango Breeding Program collection, were obtained from [35]. Sequence data were deposited in the National Center for Biotechnology Information (NCBI) database under BioProject ID: PRJNA1175065. The panel was phenotyped for FW, BC, FF, TC, and TSS. FW was measured as the average weight of 10 fruits before ripening. BC was scored as no blush, orange, pink, red, or burgundy. The hue angle was calculated using a Konica Minolta spectrophotometer using the following formula [35]:


$$ h={\mathit{\tan}}^{-1}\left(\frac{b}{a}\right) $$

In which b^*^ represents the yellowness and a^*^ represents the redness; FF was assessed with an analogue firmness meter, applying a 50-g load for 30 s, with a reported value being the average of 10 fruits. TC was calculated by measuring the scion 10 cm above the graft on trees that are 12 years old. TSS was assessed on the BRIX scale as an average of 10 fruits. More details can be found in [35].

### US data

The studied germplasm collection comprised 161 mango trees conserved at the Subtropical Horticulture Research Station, US Department of Agriculture, Agricultural Research Service, Miami, FL. These trees were selected from a larger set of 269 individuals based on their genomic and phenotypic variation. Phenotypic and genotypic data were previously published in [34] and sequencing data were deposited in NCBI under BioProject ID: PRJNA1132966. A total of 14 morphological and fruit quality traits were measured, including TSS, pulp percentage (Plup), fruit length (FLen), thickness (FThck), weight (FW), and width (FWid), and stone (StLen, StThck, StW, StWid) and seed (SeLen, SeThck, SeW, SeWid) dimensions. The pulp percentage was measured as the ratio of fruit weight to stone weight. TSS was assessed on the BRIX scale, in which one BRIX unit equals 1 g of sucrose in 100 g. More details can be found in [34].

### Chinese data

The dataset comprised a total of 224 mango trees of global origin, maintained at the breeding and preservation unit, of the South Subtropical Crops Research Institute (SSCRI) in Mazhang District, Zhanjiang City, Guangdong, China. The phenotypic and genotypic data were previously published in [26], and sequencing data was deposited in the National Genomics Data Center (NGDC) under BioProject ID: PRJCA025449. The panel was phenotyped for FW. Unlike the previous two studies, FW was measured when the fruits reached commercial maturity as the maximum weight of at least 15 fruits per tree.

### Sequencing data analysis and SNP calling

Raw paired-end sequencing reads were downloaded from the NCBI and NGDC databases and initially subjected to quality control and trimming using fastp (V0.24.0) software [69]. This process included adapter detection, removal of low-quality bases (Phred quality score <30), and filtering out reads shorter than 50 bp. Cleaned reads were aligned to the mango reference genome Irwin [70] using Bowtie2 (V2.4.5) in end-to-end alignment mode and default parameters [71]. SAMtools (V1.15) software [72] was used to convert alignments to BAM format. Only successfully mapped reads (excluding unmapped reads) were indexed for efficient access and retained for downstream analysis.

SNPs were called using BCFtools (V1.15) software [72]. First, bcftools mpileup was used to generate genotype likelihoods across all samples, referencing the Irwin genome. Subsequently, bcftools call was employed with the multiallelic and SNP-only calling model to identify variants. The raw VCF file underwent stringent filtering using VCFtools (V0.1.16) to retain high-quality SNPs [73]. Filters applied included: a maximum missing data rate of 50, a minimum minor allele count of 2, a minimum quality score of 20, and a minimum read depth (DP) of 5. The filtered VCF file was then recoded and compressed, and its index was generated using bcftools index. SNPs were further filtered with PLINK (V 2.0) to remove SNPs with >20% missing data, as well as removing singletons [74].

Imputation and phasing were performed using Beagle (V 5.5) software [75] to fill missing genotype data and infer the haplotypic phase of the genetic markers. This process is crucial for generating a complete and accurate genotype dataset, which enhances the power of downstream analyses such as GWAS and GP [76]. The software was run with its default parameters.

### Genetic diversity

To assess the genetic diversity and population structure within the integrated mango panel, two complementary approaches were employed: PCA and admixture analysis. PCA was performed using PLINK (V2.0) software [74] to visualize genetic relationships and identify major axes of variation among individuals, effectively illustrating population stratification. Subsequently, admixture analysis was conducted using the ADMIXTURE (V1.3.0) software [77]. This analysis inferred the ancestral proportions of individuals across a range of assumed ancestral populations (K), with the optimal K value determined through 20-fold CV. For each tested K value, ranging between 2 and 20, 50 independent replicates were performed. The analyses were run with a specified random seed to ensure reproducibility and independence across different replicates. The optimal K value is usually determined when the average CV value starts to fluctuate. However, CV error continued to decrease monotonically in highly admixed populations, potentially leading to the selection of an excessively high *K* value. Therefore, the optimal K value was determined at the point when the CV values across most of the replicates for the same K varied significantly [78, 79]. The selected *K* represents the highest level of ancestral resolution before individual assignments become unstable. It is critical to note that the resulting discrete ancestry proportions were used solely for the visualization and descriptive analysis of genetic diversity. These K clusters were not incorporated as fixed covariates in our downstream GWAS and GS analyses.

### Linkage disequilibrium decay

LD was assessed using 10 000 SNPs randomly selected from each chromosome. LD was inferred by calculating the pairwise *r^2^* statistic within each chromosome among all SNPs using the phased LD method implemented in PLINK (V2.0) software [74]. To visualize the decay of LD, the *r^2^* values against the genetic distances between each pair of SNPs were plotted. LD decay was calculated for each dataset independently and for the combined data of 610 individuals. To estimate a critical *r^2^* value, above which LD was assumed to be indicative of true genetic linkage, a subset of 1000 SNPs per chromosome was randomly selected. Then, the *r^2^* for all SNP pairs located on different chromosomes (i.e. unlinked SNPs) was calculated. The 99th percentile of these unlinked *r^2^* values was then designated as the threshold.

### SNP-based heritability and genetic correlation

The estimation of genetic parameters, including SNP-based heritability and genetic correlations, was performed using the Genomic Restricted Maximum Likelihood (GREML) approach for each trait/environment, or pair of traits for genetic correlation, independently. GREML is a statistical method commonly applied in quantitative genetics to estimate variance components from genomic data. First, a genomic relatedness matrix (GRM) was constructed to quantify the genetic similarity between all pairs of individuals. This GRM was calculated following the methodology described by [80] and implemented within the GCTA software [81].

Subsequently, SNP-based heritability (*h^2^*) for each trait was estimated. This parameter is equivalent to narrow-sense heritability and represents the proportion of phenotypic variation attributable to the additive effects of common SNPs. The *h^2^* was calculated using the MTG2 software [82]. Furthermore, genetic correlations between traits were determined using a bivariate REML model, also implemented within MTG2. This allowed us to quantify the extent to which common genetic factors influence two different traits. To ensure the reliability of these genetic correlation estimates, traits were only included in the bivariate analysis if their estimated heritability had a standard deviation that did not exceed the heritability estimate itself.

### Genome-wide association study

While single-trait GWAS analyses were previously reported for the individual datasets [26, 34, 35], FW and TSS were reanalyzed using the integrated dataset. This reanalysis aimed to enhance GWAS power by leveraging the significantly larger combined panel size. To account for variations across the datasets, the dataset ID was fitted as a fixed covariate in the model. A mixed linear GWAS model was implemented in GEMMA (V 0.98.5; [83, 84]) to identify QTLs associated with FW and TSS. Only SNPs with a minor allele frequency >0.05 were included in the GWAS. To establish a robust significance threshold, first, the GEC (V 0.2; [37]) software was used to estimate the effective number of independent SNPs within the panel. This step is crucial because it accounts for LD, preventing an overestimation of the significance threshold that can occur if SNPs are assumed to be fully independent (as in a standard Bonferroni correction). The final GWAS significance threshold was then calculated using the Bonferroni correction by dividing 0.05 by this estimated number of independent SNPs.

### Genomic prediction

GP accuracy was evaluated using five different models. The ‘single’ model analyzed each trait (single trait/single environment) independently. The ‘EG’ model considered FW and TSS traits for all environments together without accounting for genotype-by-environment interaction. The ‘GxE’ model incorporated GxE interaction. Additionally, a multitrait ‘MT’ model was used to analyze multiple correlated traits simultaneously, and an ‘MxT’ model that included interactions among these traits. For the 20 individuals that had clonal relation between the Australian and the US collections (Table S6), the GP analysis was conducted twice, first considering them as completely different individuals, and second considering them as the same individuals repeated over different the two environments. However, because the results were highly consistent in both analyses, only the first scenario was reported in the paper.

The general GP equation used across these models was [85]:


$$ y=\mu +E+G+T+ GE+ GT+\varepsilon $$

Here, y represents the observed phenotypes, μ is the overall intercept. **E** denotes the environmental effect, assumed to follow a normal distribution, **E** ~ N(0,${V}_E{\sigma}_E^2$), where ${V}_E$ is calculated by multiplying an incidence matrix ${Z}_E$ by its transpose. **G** represents the additive genotypic effects, following a normal distribution, **G** ~ N(0,${V}_G{\sigma}_G^2$). In which, ${V}_G={Z}_G Grm{Z}_G^{\hbox{'}}$, where ${Z}_G$ is the incidence matrix and ***Grm*** is the genomic relatedness matrix [86]. **T** denotes the trait effect in the MT and MxT analyses, assumed to follow a normal distribution, **T** ~ N(0,${V}_T{\sigma}_T^2$), where ${V}_T$ is calculated by multiplying an incidence matrix ${Z}_T$ by its transpose. **GE** accounts for the GxE interaction following the normal distribution **GE** ~ N(0,${V}_{GE}{\sigma}_{GE}^2$); in which ${V}_{GE}={V}_G\odot{V}_E$, where $\odot$ denotes the Hadamard (elementwise) product. **GT** accounts for the genotype-by-trait interaction following the normal distribution **GT** ~ N(0,${V}_{GT}{\sigma}_{GT}^2$); in which ${V}_{GT}={V}_G\odot{V}_T$. And $\varepsilon$ ~ N(0,${\sigma}_{\varepsilon}^2$) represents the independent and identically distributed (i.i.d.) residual errors.

Specific models used in the present studies are described in Table 4. These models, incorporating the relevant matrices, were fitted using Reproducing Kernel Hilbert Space (RKHS) regression, implemented in the BGLR R package [87–89]. Each analysis involved 25 000 burn-in iterations, followed by an additional 25 000 iterations for parameter estimation.

**Table 4:** Summary of the statistical models evaluated in the present study

**Model**	**Model ID**	**Equations**
**Single trait/Environment**	Single	y = μ + G + ε
**Multienvironment**	EG	y = μ + E + G + ε
	GxE	y = μ + E + G + GE + ε
**Multitrait**	MT	y = μ + T + G + ε
	MxT	y = μ + T + G + GT + ε

### Cross-validation

Two CV strategies were implemented for the GP models. These strategies were designed to simulate practical breeding scenarios, as previously described by [62]. Both approaches involved masking a subset of phenotypic data, treating it as missing, to enable the calculation of GEBVs for these masked individuals. This masked data then served as the validation set, while the remaining phenotypic records formed the reference population for training the prediction models. The accuracy of GP for each model was determined by calculating the Pearson correlation coefficient between the observed phenotypic values and the GEBVs within the validation set.

The first strategy, CV1, simulated a scenario where entirely new individuals are introduced into a breeding program. Here, phenotypic data for 20% of the individuals were randomly masked. The remaining 80% of the individuals formed the reference population. This strategy evaluates how well the models predict traits for new, unphenotyped individuals. The second strategy, CV2, mimics scenarios with unbalanced or incomplete phenotypic data across traits or environments. In this approach, 20% of random individuals were masked independently for each trait/environment. This means an individual might have phenotypic records for some traits/environments within the reference population but not for others. CV2 aims to assess the model’s ability to predict performance for all individuals across all traits from unbalanced data. Therefore, CV2 is only applicable to the multitrait model given that it is the only scenario that involves multiple phenotypic records per-single individual in the present study. Each scenario was run with 50 independent replicates, and the significant differences in prediction accuracy among different models were evaluated using Student’s *t*-tests (*P* < .05) over the 50 replicates.

## Supplementary Material

Web_Material_uhag004

## Data Availability

All data used in the present paper was previously published in [35]; [34]; and [26].
