# Supplementary figures and images for: Strategic global data integration to improve genomic prediction accuracy in tree breeding programs facing resource limitations, a case study in mango
Source: Hortic Res. 2026 Jan 6;13(4):uhag004. doi: 10.1093/hr/uhag004 (PMC13103480; doi:10.1093/hr/uhag004)

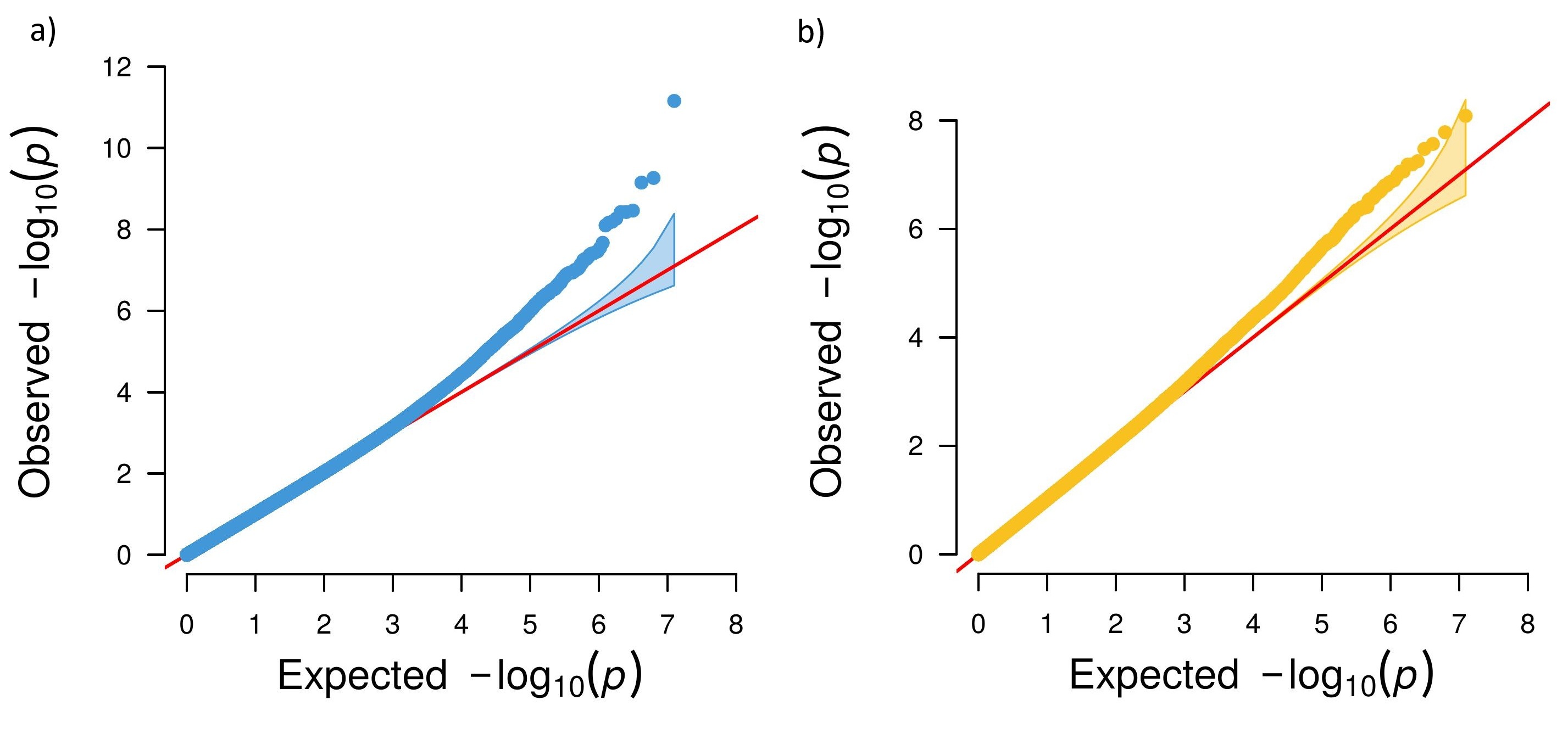

Supplement: Web_Material_uhag004 [file web_material_uhag004.zip › FigureS1.jpg]
